# Annotations

**Published:** 1889-03-16

**Authors:** 


### ANNOTATIONS.

**From the Court to the Hospital.**

MOST women now and then read the accounts given in the newspapers of the Queen's Drawing-room, and the dresses worn there. Their imagination finds a pleasure in the mental picture of these diamonds and brocades, they almost see the state and richness of the scene, they hear the rustling of the silks, and perceive the odour of the fragrant exotics. Bouquets are as large as ever, we are informed by the papers that pay most heed to these matters, and if possible more lovely. Roses of every shade, eucharis, lilies, and the rarest orchids, and—rather than the homely daffodil which Shakespeare praised—"take the winds of March with beauty." What becomes of these flowers? We know that the jewels will shine out again on other State occasions, that the dainty gowns will be seen again at balls; but what is the destiny of the Drawing-room bouquets during the remaining day or two of their fragrant life? Perhaps the débutantes put away a sprig or two of lily of the valley or azalea, as brides do the orange blossom they wore at their wedding, to look at in the after-days; but it is to be feared that many of them are cast aside, and the owners who carried them in their first freshness a few hours before, know nothing more about them. Yet it is a pity that these flowers should be allowed to fade and die after having given pleasure only for so brief a space. Flowers are such precious things, these "stars, which in earth's firmament do dwell," have such power to cheer and brighten lives on which the night of pain has fallen, that it seems a pity that any gleam of their colour, any atom of their fragrance should be wasted. We make an appeal to our ladies. These bouquets, which are of no account in your eyes after an hour or two, would give a pleasure you can hardly guess to the patients in our hospitals and infirmaries. Might not the flowers whose first freshness was given to the presence-chamber of Royalty be bestowed on those to whom has come the consecration of pain, who are perhaps about to attain the inalienable majesty of death? It would be easy for ladies in quitting the palace to leave their bouquets behind, in some cloak-room set apart for the purpose, if it were too much trouble for them to send them to a hospital. Our Queen would surely not grudge the attendance of one or two of her servants to take the flowers to suitable institutions; and—call it snobbery if you will, though loyalty would be a fitter name—they will be the more valued by the sick and the poor because there is the chance that a few hours before, her eyes had glanced at their fragrant petals.

**The Brain as a Working Tool.**

"MAN is a tool-using animal." With increasing complexity of surroundings there has usually been associated in his history a developing intelligence, and the production of more elaborate and more useful tools. The environment of modern civilised man is becoming exceedingly complex, and the conditions of survival seem to be attaining a maximum of difficulty. It is a practical question of deep significance whether any existing race of men has the staying power needed for indefinite progressive development. In this country, although average life is prolonged, there appears to be no present improvement in physique. If there be no advance in general physique, is there a compensating development of brain power? That seems to be the fundamental and urgent question of the day. That there is an all-devouring industry, that there is tremendous will-force in a few individuals, that there is a resistless ambition which drives men to distinction or to destruction, no one who has eyes can for a moment doubt. But is the average man's brain, as a thinking, planning, purposing, guiding, and enduringly-propelling instrument, improving or retrograding? Of all the tools that man possesses for his life-work, of all his weapons of defence amid an environment full of dangers, the brain is first and chief. Is this brain improving? It is becoming more active, but is it at the same time becoming stronger, tougher, more capable of continued exertion, more alert, a better judging instrument, a firmer deciding authority? Mere activity does not necessarily mean either strength or capacity. Mere obstinacy does not mean foresight and staying power. Are the brain activities and the general methods of life to-day of such a character as to develop permanent strength and capacity, or is their intensity of that unmeasured kind which overshoots the mark, and leads to premature exhaustion in the present generation, and still greater enfeeblement in the next? It seems indubitable that if the brain of the modern man be the real goose that lays the golden eggs, many of us are fast killing it; if it be the tool of all others which is absolutely indispensable, many of us are grinding it so fiercely and often, in order to keep it sharpened, that the tool itself will soon be ground away. Modern Britons, as a race, need both to look ahead and see what future conflicts they are entering upon, and also to measure their resources. The human brain is ultimately to conquer the world. So much appears to be certain. But is it to be the British or any other brain now existing, or will a new race need to come to the front in order to enter, upon the broad and ample conquests which lie before us? With us there are still some stores of undeveloped energye and if we have skill to use these wisely, we may yet be the coming race. Calm sense and patience can alone steer the ship safely. Blind hurry is sure to dash her upon the rocks.

**"Common Humanity" in Lancashire.**

"HERE, take these men." The men were two in number, both very ill, and one of them dying. The speaker was an official of the Bootle Borough Hospital, and he had brought the dying man and his companion in a cart or a cab from the hospital ward to the office of the relieving officer, in order to get rid of them and of all responsibility connected with them. Both the men, it is perhaps needless to say, were paupers, and if either or both of them had died they would have had to be buried, and that at the cost of the Bootle Borough Hospital. To prevent such a calamity as the payment of funeral expenses of two paupers they were taken out of the hospital ward, though one of them was then dying, and hurried off in a cart or cab, not to another hospital, but to the relieving officer, who might do what he pleased with them—even leave them to die in the street at his door, so far as the officials of the Bootle Hospital were concerned. These facts are reported in the *Liverpool Evening News*. What can there be in official life which so hardens men's hearts and enfeebles their brains? It is hardly likely that the house surgeon at Bootle is a born fool and brute; the matron is probably a woman of average kindness of disposition; the nurse and the porter may be presumed to be not absolutely destitute of ordinary human feeling. Yet here are three or four people who from their position ought to be expected to feel and display a maximum of human kindness, who presumably unite and concur in dragging a dying man out of his bed, bundling him off to the relieving officer's door, and leaving him there helpless and all but dead. The thing is incredible. Would not any one of these officials, even the very porter, have given the pound or thirty shillings which the pauper funeral might have cost, to the dying man, if his imagination had been properly appealed to? If the doctor had been treating the very same man in the hospital ward for diphtheria, would he not if necessary have sucked out the tracheotomy tube at the imminent risk of his own life? What startles thinking persons in cases of this kind is not merely the want of humanity, though that is shocking enough, but the imbecile want of sense. Have the Bootle officials not imagination enough to understand that even a pauper by his death atones for his pauperism and becomes sacred to all mankind? Lancashire people are often credited with being rough in manner, but they are not generally understood to be brutal in feeling. The hospital authorities owe it to themselves to explain the circumstances to the public, if explanation be possible; and if not they should visit the disgraceful behaviour of the responsible official with such signal marks of their displeasure as will not only preserve him or her from like acts in future, but will furnish a needed warning to official minds all the district over.

